# Development and pretesting of an information, education and communication (IEC) focused antenatal care handbook in Pakistan

**DOI:** 10.1186/1756-0500-4-91

**Published:** 2011-03-30

**Authors:** Saima Akhund, Bilal Iqbal Avan

**Affiliations:** 1 Department of Community Medicine, Dow University of Health Sciences, Karachi, Pakistan; 2 Immpact, Division of Applied Health Sciences, University of Aberdeen, UK; 3 Human Development programme, Department of Community Health Sciences, Aga Khan University, Karachi, Pakistan

## Abstract

**Background:**

Improvement of maternal morbidity and mortality indicators remains a major challenge for developing countries. Antenatal care is one of the key strategies in maintaining safe motherhood. The objective of this study was to develop and pretest a culturally relevant Antenatal Care Handbook (ANC handbook) utilizing the principles of information, education, and communication (IEC). We developed the ANC handbook after an extensive review of existing literature, available instruments (for keeping track of pregnancy and informing pregnant women), and seeking expert opinion. To pretest the ANC handbook, a cross-sectional approach was adopted, and information was collected from 300 expectant women, 150 women each from the community and from the health facility arm. Trained field workers conducted the pretesting from May 2004 to June 2004. Feedback on messages for pregnant mothers contained in the handbook was also assessed. At the same time, the ANC handbook was reviewed by 25 health care providers (including community health workers, physicians, nurses, and other health staff working at various kinds of health care facilities). Data were analyzed using both quantitative and qualitative methods.

**Findings:**

Twenty-three percent of the interviewed women were primigravida, 50% were multigravida and 27% were grandmultipara. The mean age of the women in the community sample was 25.8 SD: 4.9 years and in the hospital sample it was 25.7 SD: 5.2 years. No significant differences were observed between women interviewed at community or health facilities related to their understanding of ANC messages, and the majority of messages were well understood. Similarly, health care providers found all of the instruments useful and workable in the health system. Finally, feedback from pregnant women and health care staff regarding different components of the handbook were incorporated and later verified by them.

**Conclusions:**

Findings of pretesting reveal that a majority of pregnant women have an understanding of the culturally relevant ANC handbook. The handbook was found to be practical by healthcare paraprofessionals and community workers for use in different tiers of the health care system in Pakistan. The ANC handbook can be applied in the health service sector of Pakistan and can be adopted with relevant cultural modifications by countries with a similar context.

## Background

Of the estimated total of 536,000 maternal deaths worldwide, developing countries accounted for 99% of the deaths in 2005 [1]. This difference in pregnancy associated risk is often considered the greatest health divide between the developed and developing world. It is widely agreed that addressing this gap requires broad intersectoral interventions. However, at a minimum every women must be ensured access to maternal health care services comprising of antenatal care, emergency obstetric care, and postpartum care [2].

The existence of antenatal care and education programs is well established in developed countries, and consequently, they have been instigated in developing countries as well [3-7]. Despite some controversy over the effectiveness of antenatal care [8,9], it has remained a key strategy for reducing maternal mortality [10-14].

Antenatal care consists of care provided to women during pregnancy by skilled health personnel [15]. It includes health assessment of pregnant women, encouraging good health habits, addressing pregnancy related complications and providing social and psychological support [15,16]. Although ANC alone cannot directly bring reductions in maternal mortality, its potential value as an entry point for expectant mothers into the health system as well as increasing the rates of deliveries assisted by skilled provider is well recognized [17,18]. Behaviours related to infant care and contraception are also found to be influenced by antenatal care [19,20].

Research has identified a range of individual, household, and community level factors that can influence utilization of antenatal care by women. The individual level factors mainly include low educational status, autonomy, decision making power, and lack of awareness of the need for routine care visits even in the absence of danger signs [21-24]. A woman's recourse to antenatal care is also conditioned by certain factors such as household income, ethnicity, and lack of transportation [25,26]. Some of the community level factors affecting antenatal care utilization include geographic location of community, existence of a functioning health facility, distance to the referral level facilities, and presence of a health worker providing antenatal care [4,27-30]. It is apparent that those seeking ANC may exhibit a vast array of complex behaviours. The ways in which these intricate behaviours translate to individual actions can be largely influenced by cultural norms and traditions [31,32]. Consequently, several communication strategies for better utilization of antenatal care services by women were used such as the use of video films, radio and television commercials, women groups, community groups, and positive deviance reinforcements etc. [33-35]. One of these several strategies is the development of Maternal and Child Handbook system used in Japan [36,37]. An important feature in this connection is that each country tried to develop their own version of the Maternal and Child Handbook to suit local cultural norms and the available health services. Examples of countries which have adapted the ANC handbook system include Indonesia, Palestine, Korea, Vietnam, and Thailand [38-40].

We describe here research that was conducted in Pakistan against the backdrop of a high maternal mortality ratio (276 per 100,000 live births) and low antenatal care uptake (28% women complete WHO recommended four antenatal care visit model) [41]. Despite decades of intervention funded by state and international agencies, poor maternal health care indicators not only point toward fundamental macro-level issues such as poverty, illiteracy, and low status of women, [42] but also show shortcomings in the design and delivery of these programs [43,44]. Consideration of education and communication aspects is vital in the delivery of health interventions [45]. Previous research reports the importance of antenatal contacts on women's decision to deliver at health facilities and thereby propose antenatal care programs to focus upon the education and communication content [24]. It has been argued that making use of an instrument or educational material developed in an industrialized country and imposing it on culturally diverse settings can seriously limit the validity of results as well as benefits of an intervention [46]. Hence, we decided to address some of the issues related to design and delivery of antenatal care interventions through the development of an antenatal care handbook (ANC handbook) using information, education and communication (IEC) focused principles.

Conventionally, the IEC approach is used in the field of reproductive health for creating awareness, increasing knowledge, changing attitudes and moving people to change their behaviour or adapt an innovation [31]. The successes of IEC strategy at various national, sectoral and programme level behaviour change interventions are well documented [47]. WHO recommends the following framework principles while developing, implementing, and evaluating IEC interventions [31]:

▪ Clear objectives

▪ Client centeredness

▪ Appropriate research methodology

▪ Emphasis on positive behaviour change

▪ Carefully crafted and tested educational messages

▪ Appropriate channels of communication

▪ Use of inexpensive educational materials

▪ Culturally relevant graphic messages for home use

▪ Linkage with health care delivery system

▪ Mechanisms for monitoring, evaluation and feedback

The ANC handbook was developed utilizing the above mentioned IEC principles. It was constructed while keeping the following purposes in mind: to educate pregnant women about their own health through an organized set of antenatal care related messages, to emphasize the importance of keeping a record of pregnancy by the women, to increase health worker capacity to help pregnant women in adapting positive behaviour according to each trimester of pregnancy and to link them with the health system.

The need for development of a new ANC handbook was felt because although a variety of antenatal care recording and education delivery instruments were available at varying levels of health facilities in Pakistan, these were either too complicated for use at the community level (these were the cards mostly used at secondary and tertiary level care facility, and missing or deficient in educational aspects), or were oversimplified, hence lacking technical accuracy. No information regarding their validation in the local context was available. Many instruments meant for community based antenatal care were not available in the local language. Various adaptations of the antenatal card recommended by government were in use by various health facilities, but they were lacking different elements, such as the educational component for pregnant women [48]. To further complicate matters, there was a lack of integration of antenatal care instruments to be used at different levels of health facilities. For these reasons, a document for maternal care was developed with the potential to record and guide continuity of care right from the household up to the tertiary level care facilities.

The first part of this paper describes the process of development of an IEC based ANC handbook. The second part delineates the process of pretesting the ANC handbook both in terms of practicality in the existing health system and understanding and acceptability by the main stakeholders (i.e. antenatal care service providers and users) in Karachi, Pakistan.

Karachi is the largest port city of Pakistan as well as the cultural, economical, educational, and political centre. Karachi is home to more than ten percent of Pakistan's 160 million population who have migrated and settled mainly due to economic and educational opportunities [49]. The reason for conducting pretesting of the ANC handbook in Karachi was its diverse mix of the population which represents all major ethnic and socioeconomic groups, the availability of a wide range of health care facilities and for feasibility considerations.

## Methods

Methods for the study can be broadly explained under two headings according to objectives of the study:

a) Development of the ANC handbook

b) Pretesting of the ANC handbook

### a) Development of the ANC Handbook

The first step in developing the ANC handbook involved literature review regarding theoretical framework of different components of antenatal care, the type of different antenatal care record keeping, and information giving instruments/materials available at the community and health facility levels in Pakistan.

The ANC handbook is comprised of two major sections, i.e. the *Pregnancy Record Card (PRC) *and the *Pregnancy Education Card (PEC)*. The PRC was developed for monitoring the health of pregnant women during the course of pregnancy. The inclusion of items comprising the PRC was based on the theoretical understanding of antenatal care from a biomedical perspective. It contained variables to record the history of the past and present pregnancies, information regarding clinical tests, medications, and other relevant aspects pertinent to pregnant women.

The PEC was developed for encouraging good health habits, providing health education, and offering support to pregnant mothers. The content of the PEC was composed of pregnancy, child birth, and child spacing related messages which were accompanied by culturally appropriate sketches. Message specific illustrations were designed by a graphic artist.

In order to ensure that each component of the ANC handbook was administered and recorded in a predetermined and consistent way, both the PRC and PEC were accompanied by their corresponding manual of instructions. *The Manual of Instructions for PRC *contained instructions for understanding as well as administration of each of its items, its purpose, and method to administer and record. It also included helpful hints in case of difficult variables. For example, if a health worker has to ask the date of the last menstrual period from an expectant mother, she would learn its *purpose *in calculating the stage of pregnancy, and also in estimating the expected date of delivery so that delivery related arrangements can be made. The *recording *instruction would ask her to note the date in terms of day, month, and year format. The associated *helpful hint *would guide her to relate it to local calendar and specific religious/cultural events that have taken place recently if the expectant woman doesn't remember the exact date of the last menstrual period. *The Manual of Instructions for PEC *provides detailed information related to an educational message so that if expectant women have any difficulty in understanding a particular message or wants to learn more about it, the health worker should be able to do so.

For making the ANC handbook presentable, convenient, easily understandable, and succinct, a detailed workup on the finalization of the layout was carried out. After the title page, the first section of the ANC handbook is the PRC followed by the PEC which was arranged in a pregnancy trimester specific manner. The number of pages of the PEC were three (i.e. a single page specific for each pregnancy trimester). This was done in the light of the evidence that trimester specific messages are more easily understood by women with low literacy and socioeconomic status as it reduces the amount of information while also making it relevant for the immediate period of pregnancy [50].

Once the PRC, PEC, and their respective manual of instructions were ready, these were then translated into the national language (Urdu), which is widely spoken and understood in the country. The translation of the handbook components back into English was done by independent researchers.

#### Intended operationalization of the ANC handbook

The ANC handbook was developed to be utilized by frontline community health workers (such as Lady Health Workers and Midwives) as well as facility based Lady Health Visitors and other senior health staff. The uniqueness of the ANC handbook is that it can be used uniformly across different tiers of the health system in Pakistan; whether community based or hospital based. The intended operationalization of the ANC handbook is that the expectant mothers will keep a copy of PRC and similarly, a copy will be retained by staff at the nearest public/private health facility. Hence, after examining the expectant mother, the staff will be able to record their findings on both copies of the record. The copy with the expectant woman will ensure quick availability of obstetric history in case of emergency besides creating a persistent awareness of importance of woman's own as well as the baby's well-being. The copy at the health facility will ensure the availability of pertinent information in case the expectant woman's copy is lost and will also form an important component of pregnancy related statistics. During the same visit when pregnancy related technical information will be recorded, reinforcement of the information contained in the PEC will also be carried out (a sample page from PEC is given as additional file 1).

#### Pilot testing

A small scale pilot test was undertaken as recommended by van Taijlingen and Hundley [51] to check if the developed instruments have any unforeseen problems such as wording or flow of the items, as well as to see if the proposed methodology is feasible. Pilot testing of the ANC was carried out using the same techniques as were used to test the instruments in real settings. PEC was administered to 15 pregnant women at a private health facility (that was not included in the study conduct sites) along with 5 obstetricians and nursing staff each.

### b) Pretesting of the ANC Handbook

In order to determine whether the expectant mothers found the sketched message illustrations in the PEC to be culturally appropriate and relevant, and whether the health staff working at different facilities found it workable in the health system, we adopted a cross-sectional approach. Traditionally, it is considered valuable to divide pregnancy into three equal parts called trimesters, each trimester being three months long. This classification identifies the important obstetrical milestones easily [15]. Hence, it was essential to include a sample representative of all trimesters.

**Figure 1:**
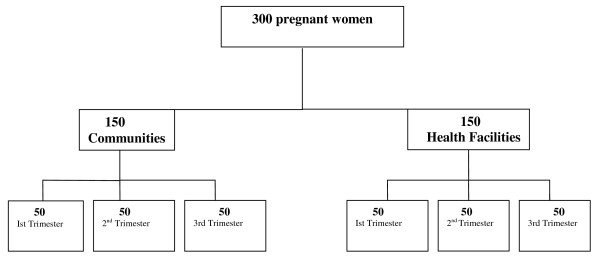
**Sampling strategy for field testing of Antenatal Care Handbook in Pakistan**.

A convenient sampling strategy was adopted because no sampling framework of pregnant women was available in the study communities. Our total sample was 300 pregnant women with equal representation from each pregnancy trimester. Keeping in view the potential differences among the pregnant women who utilize antenatal care and those who don't, we recruited 150 women from the community arm and 150 from the health facility arm. Each arm is further divided to include 50 women from all pregnancy trimesters to cover the entire spectrum of pregnancy (see Figure 1).

A team of eight female data collectors was recruited. These included sociologists, midwives, and other field staff who have past experience in doing field research related to maternal health. They were given two days training in which all the components of the ANC handbook were described in detail. The selection criteria, procedure for taking informed consent, and other data recording methods were explained in detail.

#### Data collection

Our data collection period lasted from 06 May to 05 June of 2004, and it included both the health facility and community arms. The pretesting of the two components of the ANC handbook was carried out in the following manner:

### Pretesting of PEC

#### Health facility arm

For the achievement of the sample needed from the health facilities, three primary health care centres (namely Sultanabad, Hijrat Colony, and Rehri Goth), one government maternity home (PIB colony), and an outpatient department of a tertiary care hospital from Karachi (Community Health Centre-Aga Khan University Hospital) were used as study sites. The selection of these sites was based on the diversity of population they were serving, presence of a functional antenatal care service, and time/logistical constraints. The eligibility criteria encompassed pregnant women visiting the centre for antenatal check up and who gave informed consent verbally. The first page of the study questionnaire contained a standard paragraph regarding the purpose of the study, assurance regarding confidentiality of the participants' information, and voluntary participation (if the women had any questions regarding participation in the study then these were also answered by the field workers). It further included a statement regarding whether the study participant had consented to participate in the study. The field workers read out the standard paragraph to the pregnant women, and asked for their voluntary participation in the study. If the woman agreed for participation in the study, the questionnaire was marked and the interview proceeded. After the collection of information on socio-demographic variables, and, past and current obstetric history, the women were given PEC to review for some time on their own. When they finished reviewing the material, the field worker asked their understanding of each sketch and accompanying health education message. The results were noted as 'understandable', 'not understandable' and 'comments'.

#### Community Arm

Data were collected from the squatter settlements of Manzoor Colony and Qayumabad, Karachi for the community arm portion of the study. These sites were selected as they present a good mix of major ethnicities of Pakistan with inhabitants mostly belonging to low socioeconomic status with high levels of illiteracy and poverty. An adequate mix of public and private antenatal care providing facilities was also present at these field sites.

Pregnant women were identified by doing door to door mapping. The selection criteria encompassed pregnant women who were not receiving antenatal care for the current pregnancy, were not planning to seek routine antenatal care, and who gave informed consent verbally (the process of taking and recording the informed consent was same as described under the heading of health facility arm). During data collection, the field workers collected background sociodemographic, past and current obstetric history related variables in the questionnaire. In order to determine its understanding by the target population, the sketched illustrations and accompanying health education related messages were given to the expectant mothers. The opinion of the expectant mothers regarding sketched illustrations and health education related messages was noted in the questionnaire as 'understandable', 'not understandable', and 'comments'.

### Pretesting of PRC

Owing to the technical nature of the contents of the PRC, its field testing was carried out by presenting it to health care staff of different levels of health facilities. The staff of the health facilities mentioned in the hospital arm: 3 primary health care centres (Sultanabad, Hijrat Colony, and Rehri Goth), one government maternity home (PIB colony) and an outpatient department of a tertiary care hospital from Karachi (Community Health Centre-Aga Khan University Hospital) participated in testing of PRC. They were given PRC one day prior to collecting data from them so as to give some time for content review and also to minimally affect their routines at the clinics. On the day of pretesting, after doing antenatal assessment of the expectant women, the health care staff recorded the information on PRC to check its completeness and user friendliness. A total of 25 health care staff participated in the pretesting of PRC. These included Community Health Nurses, Midwives, Health Technicians, Registered Nurses, Lady Health Visitors, General Physicians and Obstetricians who were working in the primary health centres, maternity homes and hospitals.

### Quality Assurance

The data collection team was supervised in the field by the authors. Their tasks included accompanying the field workers and observing them, cross checking the collected data, clarification of ambiguities, and the identification of missing information. Data were edited in the office for consistency, accuracy, and identification of the out of range entries (for example mention of past obstetric history related variable if the respondent was a primigravida). In case of missing information, the field workers were advised to go back to that particular study participant and complete the required information. For data validation, double entry was done in MS access, and to identify any inconsistency in data entry, 5% of the records were re-entered.

### Ethical Considerations

Ethical review and formal permissions for the conduct of study were taken from all the participating institutions according to their specific protocols, including the ANC handbook which was shared with the management of the health facilities in advance. Informed consent was taken from all study participants. Involvement in the study was voluntary, and in case of refusal, the participant's decision was respected. The refusal rate was less than 1 percent of the total sample in the community as well as health facility arm. The study participants who were identified as having high-risk conditions were referred to seek expert advice. The messages given in the 'PEC' were reinforced. Field workers were trained not to disclose study participants' information with any other person in order to maintain confidentiality. Data were entered and analyzed in an anonymous manner so as to protect the identity of the study subjects.

Data were analysed using quantitative and qualitative techniques. Quantitative analysis was done using SPSS version 14.0. Mean and standard deviation of continuous variables such as age, and proportion of categorical variables (such as religion, ethnicity and literacy status) were calculated. Chi square test was used to compare any significant difference between women in the community and the health facility arm. A P value of <0.05 was considered as significant. Comments of the respondents regarding each message of the PEC were compiled and common themes were identified for appropriate modification.

## Results

### Sample characteristics for PEC testing

Table 1 illustrates the socio-demographic characteristics of the study sample according to their affiliation with the health facility or the community arm of the study. A majority (i.e. 98%) of women from both the study arms were under the age of 35 years. The mean age of women in community sample was 25.8 SD: 4.9 years, and in the hospital sample it was 25.7 SD: 5.2 years. An overwhelming majority of the population was Muslim in both the study groups. About 11% percent of women in the community interviews were either Christians or Hindus, versus 6% in the health facility interviews. Distribution of respondents by the level of educational attainment showed that an equal proportion of respondents (i.e. 50%) were illiterate in both the study components. With regard to ethnicity, approximately 40% of the sample group were mohajirs (ethnic group that migrated from Indian part of subcontinent to Pakistan) and the rest non-mohajirs (inland ethnic groups such as Sindhis, Punjabis, Pathans etc.). Ten percent of women in the community setting and 7% interviewed from the health facility setting were involved in paid jobs.

**Table 1:** Characteristics of expectant mothers interviewed for field testing of PEC at community and health care settings in Karachi

Variable	Community Arm (N = 150)	Health Facility Arm(N = 150)	P value
**Age (in years)**			
< 35	147(98.0)	146(97.3)	0.70
≥ 35	03(2.0)	04(2.7)	
Mean (SD)	25.8(4.9)	25.7(5.2)	
			
**Religion**			
Muslim	134(89.3)	141(94.0)	0.05
Non Muslim	16(10.7)	09(6.0)	
			
**Ethnicity**			
Mohajir	62(41.3)	59(39.3)	0.72
Non Mohajir	88(58.7)	91(60.7)	
			
**Literacy status**			
No	52(50.0)	52(50.0)	1.00
Yes	98(50.0)	98(50.0)	
			
**Employment Status**			
Yes	14(9.3)	11(7.3)	0.53
No	136(90.7)	139(92.7)	

### Past obstetric history

Table 2 depicts the obstetric history of women with regards to both current and past pregnancies. In terms of number of pregnancies, 83% of respondents were multigravida (those with 2 or more pregnancies) in the community arm and 71% in the health facility arm. Almost an equal proportion of women (i.e. 90%), had the outcome of their last pregnancy as a live birth. Forty- seven percent women delivered their last baby at home among the community interviewees and 28% among health facility interviewees, and this difference was statistically significant (P value: 0.00) as well. A higher proportion of respondents who were interviewed at the health facilities had their previous delivery assisted by a doctor than those who were interviewed in the community set up (61% and 53% respectively). However, this difference was statistically insignificant (P value: 0.47).

**Table 2:** Past obstetric history of expectant mothers interviewed for field testing of PEC at community & health care settings in Karachi

Variable	Community Arm	Health Facility Arm	P value
**Gravida^±^**			
Primi	25(16.7)	44(29.3)	
Multi	125(83.3)	106(70.7)	0.09
			
Last Pregnancy outcome*			
Live birth	100(90.0)	92(92.0)	
Abortion	3(2.7)	2(2.0)	0.00
Still Birth	4(3.5)	5(5.0)	
Neonatal deaths	4(3.5)	1(1.0)	
			
Place of delivery*			
Home	52(46.8)	28(28.0)	
Health Centre	7(6.3)	15(15.0)	0.00
Hospital	52(46.8)	57(57.0)	
			
Delivery attendant*			
Doctor	59(53.2)	61(61.0)	
Trained person	29(26.1)	20(20.0)	0.47
Untrained Person	23(20.7)	19(19.0)	

^± ^N = 300 and rest of the table represents multigravidas only

* Missing data (11% in the community arm and 6% in the hospital arm)

### Practices and attitudes related to current pregnancy

Table 3 describes the differences between the two groups with regard to attitudes and practices relating to current pregnancy. Six percent of women from the community arm and 1% from the hospital arm had not identified any person for delivery assistance. More than 60% of respondents from both arms of the study intended to adopt child spacing methods at the end of their current pregnancy. More women from the hospital arm reported taking iron and folate (60% and 69% respectively) as compared to the women of the community arm (35% and 42% respectively), and these differences were statistically significant as well.

**Table 3:** Practices and Attitudes related to current pregnancy of expectant mothers interviewed for field testing of PEC at community & health care settings in Karachi

Variable	Community Arm N = 150	Health Facility Arm N = 150	P value
**Identification of person to assist delivery**			
Trained	129(86.0)	136(90.6)	
Untrained	12(8.0)	12(8.0)	0.27
Person not yet identified	9(6.0)	2(1.3)	
			
**Identification of health facility as a place for delivery**			
Yes	110(73.3)	110(73.3)	0.21
No	40(26.6)	40(26.6)	
			
**Intention to adopt child spacing after delivery**			
Yes	97(66.9)	94(64.4)	0.65
No	53(33.1)	56(35.6)	
			
**Medications in the current pregnancy**			
**Folic Acid**			
Yes	63(42.0)	104(69.3)	0.00
No	87(58.0)	46(30.7)	
			
**Iron**			
Yes	53(35.3)	90(60.0)	0.00
No	97(64.7)	60(40.0)	
			
**Tetanus vaccine**			
Yes	46(30.6)	55(36.6)	
No	104(69.3)	95(63.3)	0.52

#### Review of PEC

The opinions of pregnant women regarding different sketches and descriptors of the PEC were recorded in the questionnaire as 'message', 'understandable', or 'not understandable' and 'comments'. This process was used in order to see the similarity and frequency of responses. After completion of data collection, the comments of the respondents regarding each message were qualitatively analyzed to identify common themes, and similar responses were put into categories. The common comments were used to clarify the ambiguities in message understanding by making appropriate graphic modifications. The messages were categorized in three main categories namely assessment, care provision and health promotion. For example, messages regarding diet, seeking help from expert, and high grade fever were understood by 100% of women in the community arm and 98%, 97% and 96% by the hospital arm women respectively. However, these differences were not significant statistically. Generally over 90% of the women understood the messages with the help of sketches and found this way of message delivery useful, as depicted by Table 4. Respondents in both arms of the study had difficulty in understanding the mud (mountain clay used in pregnancy-the pica behaviour) intake related message and some of the respondents commented as 'not to climb the rock in pregnancy'. Moreover many of the women commented that mud intake was not a very common practice. Similarly, for avoiding smoking during pregnancy, a large majority of respondents understood the prohibition but also questioned the reason for inclusion of this message because smoking is socially not prevalent among females in Pakistan (hence these messages were deleted in the post field testing version of the handbook).

**Table 4:** PEC understanding of expectant mothers interviewed at community & health care settings in Karachi

Message	Community Arm (N = 150)	Health Facility Arm (N = 142)	P value
**Assessment**			
By skilled health providers			
Yes	130(86.7)	122(85.9)	0.73
Getting measurements done (i.e. weight, blood pressure & Hb)			
Yes	147(98.0)	135(95.1)	0.16
**Service/care provision**			
Tetanus immunization			
Yes	148(98.7)	138(97.2)	0.37
Iron and folic acid intake			
Yes	147(98.0)	137(96.5)	0.42
**Health Promotion**			
Planning & identification of pregnancy			
Yes	128(85.3)	121(85.2)	0.93
Rest, sleep & avoidance of stress			
Yes	143(95.3)	133(93.6)	0.39
Nutrition			
Yes	150(100)	139(97.9)	0.07
Preparation for breast feeding			
Yes	149(99.3)	138(97.2)	0.15
Preparation for aseptic delivery			
Yes	145(96.7)	134(94.3)	0.23
Emergency care by health expert			
Yes	150(100.0)	140(98.6)	0.07
Danger signs & symptoms			
- Severe headache			
Yes	149(99.3)	139(97.2)	0.15
- High grade fever			
Yes	150(100.0)	136(95.7)	0.00
- Swelling of face, feet and ankles			
Yes	127(84.7)	121(85.3)	0.99
- Leaking of fluid or blood			
Yes	142(94.7)	132(93.0)	0.55
Mud intake			
Yes	141(94.0)	138(97.2)	0.31
Avoidance of self medication			
Yes	143(95.3)	127(89.4)	0.03
Nail clipping			
Yes	149(99.3)	137(96.5)	0.15
Bathing, hand washing and dental hygiene			
Yes	145(96.7)	134(94.4)	0.34
Iodized salt			
Yes	149(99.3)	136(95.8)	0.04
Smoking			
Yes	149(99.3)	139(97.9)	0.28
Family planning			
Yes	147(98.0)	137(96.5)	0.42

#### Opinion of health staff regarding ANC handbook

The opinions and suggestions of the different cadre staff at the above mentioned health facilities were used to field test the PRC and its accompanying observer's guide, PEC and observer's guide for the PEC. The staff at these facilities reviewed the different sections of all of the listed tools and provided valuable insights for its improvement. Regarding the observer's guide for the PRC, most of the staff suggested their comments regarding the 'helpful hint' segment. For example from the section on 'past pregnancies', for inquiry related to post partum haemorrhage, one of the community health nurse suggested addition of local term called 'chilla' for the post partum period. Similarly from the section on 'antenatal observations', the health staff suggested in the 'helpful hint' regarding iron and folic acid intake. The health staff advised us to include an instruction on the PRC to check the iron supplement packaging used by the pregnant women because sometimes iron and folic acid are contained in a single tablet. In the same way, regarding the PECs' segment on 'health promotion', one of the Lady Health Visitors suggested that the national logo of iodized salt (a hand and a pot) could be added in the sketch. In addition, nursing staff and physicians suggested better space allocation on the PRC for writing the results of various tests that are prescribed during pregnancy.

## Discussion

The ANC handbook was developed using WHO recommended IEC strategies. We started with clear objectives of developing an ANC handbook for informing pregnant women and involving health workers in the context of high maternal mortality and lack of continuity of care for pregnant women. It is recommended that for IEC interventions to be effective, these should be designed with an understanding of the target audience and their attitudes, beliefs, values, and past behaviour. Similarly, educational and socioeconomic status of the clients should be kept in mind while designing IEC interventions [30,52,53]. The ANC handbook was developed for women with no/limited literacy and lower socioeconomic status. Appropriate epidemiological research methodology was adopted for handbook development and pretesting. All the messages of the ANC handbook emphasize positive behavioural change, for example: 'identify someone trained to assist you during pregnancy and delivery' rather than don't deliver with untrained delivery assistants. Each message of the ANC handbook was carefully crafted on the basis of literature review and expert opinion. The channel of communication for this IEC based handbook was home visitation by community health workers and pictorial messages that were developed utilizing local inexpensive print materials. We used pictorial assisted guide due to low levels of literacy among females in Pakistan and the previous success of pictorial IEC material in conveying messages with clarity [54]. The feedback of pregnant women and health care providers determined the cultural relevance of the ANC handbook. Mechanisms for monitoring by health care para-professionals and pregnant women were built into the handbook system. Linkages with health system were considered and PRC section of this IEC focused handbook was meant to be completed by paraprofessionals at the nearest health facility. Moreover, local community health workers are also expected to work as a liaison between the pregnant women and the nearest health facility. The materials for home use in the form of sketched illustrations were prepared with the help of a graphic artist. The ANC handbook was evaluated by presenting it to 300 pregnant women and 25 health care providers including Community Health Nurses, Lady Health Visitors, Midwives and Physicians. Their feedback regarding different components was gathered, analysed, incorporated, and again finally verified by a sample of those who provided feedback.

### Limitations

The limitations of our study were the testing of IEC materials on an urban population. However, the composition of our study shows that our sample was ethnically diverse and many of the participants were inland migrants from the rural population of other provinces who later relocated in Karachi. Although our study communities were from low socioeconomic squatter settlements, their exposure level to antenatal care related information may possibly differ from the general population of Pakistan. This is because they may already be exposed to some of the messages due to their urban residence and hence, find messages in the PEC understandable. For pretesting of the PEC in the health facility arm, we were not able to check understanding and receive comments of eight women (5%) from the health facility arm as most of these women had time constraints. However, their background characteristics were quite similar to other women included in the survey, so we expect their responses be identical to other women who completed the PEC pretesting. The survey was cross sectional in nature; hence, we were unable to check whether the health education messages imparted through the PEC were followed.

The results on obstetric history (Table 2) indicate that the delivery place does not determine delivery provider, as more women were assisted during delivery by trained attendants. This finding highlights the accessibility or quality issue of the health services. Home deliveries certainly pose a risk to the mother and the new born if life threatening complications arise [55].

It can be argued that though information and sketches given in the PEC were meant to be used by pregnant mothers, in reality this may not happen as Pakistan is a poor country with about 23% population of its population living below the poverty line [56]. Poverty is a well known risk factor for non utilization of antenatal care [20]. It may be possible that in the poverty stricken households women may spend most of their time in earning bread for the family and hence find no time to focus on the advices given in the PEC. However, this caveat may partially be addressed as we envisage this handbook operationaliztion with the involvement of local community health workers who would visit the home of pregnant women and reinforce messages given in the PEC and would also assist women in seeking health care from the nearest health facility.

Our finding of no significant difference in terms of background characteristics and understanding of the PEC messages between the community and health facility arms of the study suggest that these messages may work equally effectively at community and health facility levels of the health system. Though community women understood the messages slightly higher than women at health facilities, most of these differences were statistically insignificant. In this study we have relied upon study participants' stated understanding of the information provided in the PEC. However, as Lambert and McKevitt caution that study participants' behaviour may differ from their stated opinions and highlight the need to use anthropology guided participant observation rather than relying only on qualitative techniques [57]. It is also possible that participants may have reported the messages of PEC as understandable and appropriate due to social desirability and thus introducing obsequiousness (the Clever Hans effect) bias in the study results [58].

There was a statistically significant difference between those interviewed at community and health facility levels regarding the use of iron-folate. More women in the health facility arm were taking iron-folate in comparison with women in the community arm. The possible reason for this difference may be the routine free provision of these supplements to the expectant women by the health facilities.

Our idea of having two copies of the ANC Handbook demands consideration. The purpose of double record keeping is different. For pregnant women it will serve as their own independent copy because in Pakistan, it is a common practice that women often change their care providers as they go to their parents' house near the time of delivery. Having two copies would ensure that details of the pregnancy would be available at the time of delivery. For community workers it will be a reminder for advice to be given related to a particular pregnancy.

It can be questioned that the ANC handbook cannot directly address major health system related barriers or increase accessibility to health care, but presumably it will perform the more important function: creation and comprehension of demand for ANC. In addition, it will provide a common reference point for health care provider and pregnant women in terms of service provision and client expectations.

In countries where a majority of deliveries take place at home, it is important to involve the community based health workers who visit homes and provide information and assistance related to pregnancy and child birth [59]. In Nepal and Pakistan, use of community health workers was found successful in improving use of maternal health care services [60,61]. Currently, the ANC handbook system described in this paper is implemented at selected field sites in Sindh and Balochistan provinces under Human Development Programme of Aga Khan University, Karachi, Pakistan. Both public and private health facilities, as well as community level health set ups were included in the pretesting of the ANC handbook, which point towards instrument feasibility in diverse health care settings. We aim to propose the ANC handbook system at policy level on the basis of a larger scale intervention, in order for the system to be adapted by the government using the Pakistan's existing network of more than 92,000 Lady Health Workers who provide outreach services to communities [62].

## Conclusions

Our study tried to explore the perspectives of pregnant mothers as well as health professionals regarding the ANC handbook. Results of the pretesting suggest that IEC based instruments for monitoring pregnancy are understood by mothers, and can be incorporated in different tiers of the health system in Pakistan. We also believe that our proposed ANC handbook system will be quite relevant for other developing countries. The nature and overall principle of ANC handbook would remain the same in such a case, but graphical modifications of the illustrations would be required to maintain cultural relevance.

## Competing interests

The authors declare that they have no competing interests.

## Authors' contributions

BIA conceptualized the study and provided critical feedback for the development of ANC handbook as well as for all phases of the study conduct, analysis and write-up. SA developed all the study instruments, trained and supervised field staff, analyzed data and drafted the manuscript. All the authors approve the final version of manuscript.

## Supplementary Material

Additional file 1 **Supplemental material**. Click here for file
