# Supplementary figures and images for: Development and pretesting of an information, education and communication (IEC) focused antenatal care handbook in Pakistan
Source: BMC Res Notes. 2011 Mar 30;4:91. doi: 10.1186/1756-0500-4-91 (PMC3076271; doi:10.1186/1756-0500-4-91)

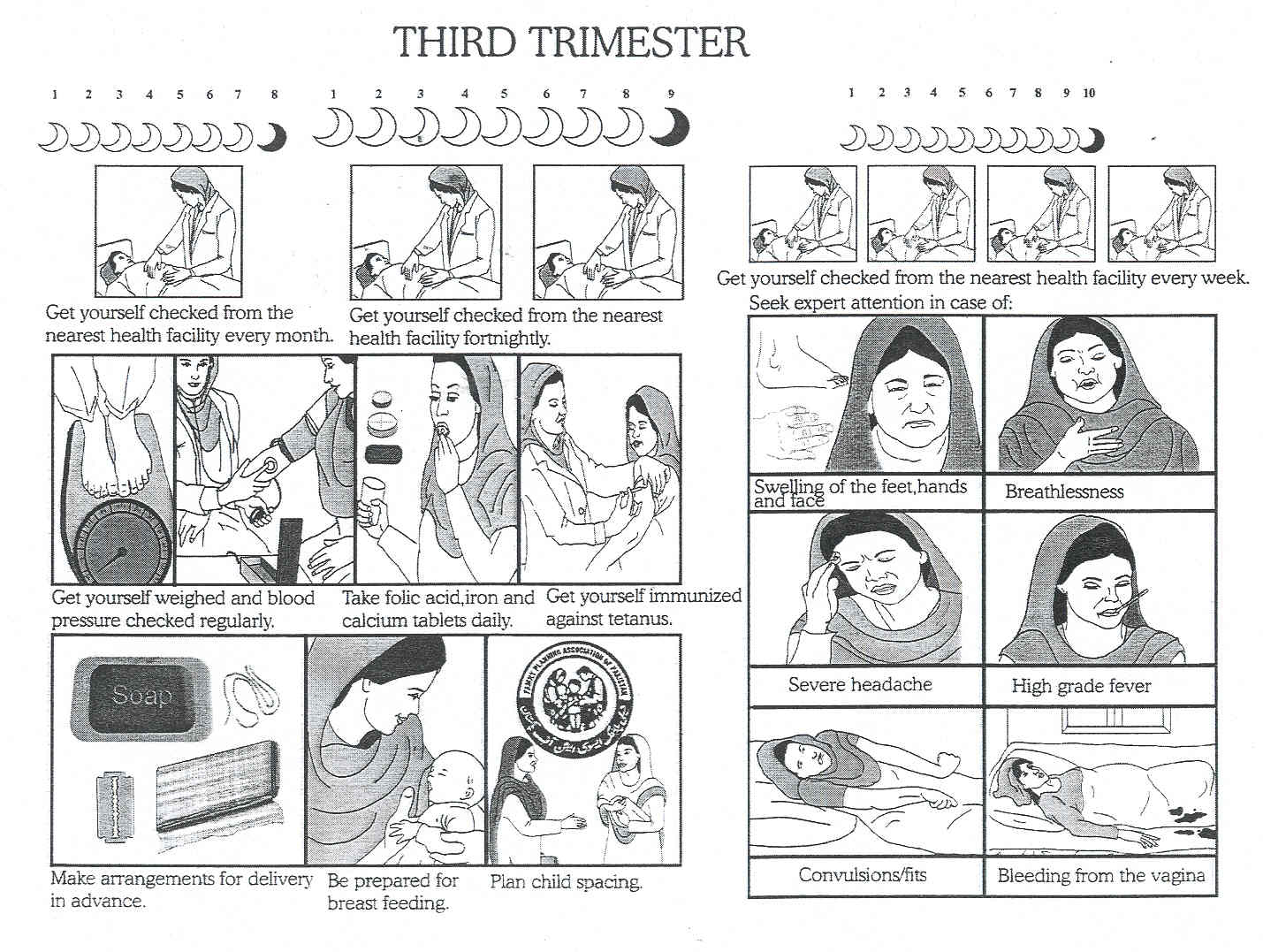

Supplement: Additional file 1 — Supplemental material. [file 1756-0500-4-91-S1.DOC]
